# Ethyl 6-amino-8-(4-chloro­phen­yl)-9-nitro-2,3,4,8-tetra­hydro­pyrido[2,1-*b*][1,3]thia­zine-7-carboxyl­ate

**DOI:** 10.1107/S1600536811032193

**Published:** 2011-08-17

**Authors:** Na Zhang, Xian Zhang, Dongmei Li

**Affiliations:** a School of Materials Science and Engineering, Shandong Institute of Light Industry, People’s Republic of China; b School of Chemistry and Chemical Engineering, University of Jinan, People’s Republic of China

## Abstract

In the structure of the title compound, C_17_H_18_ClN_3_O_4_S, the thia­zinane ring displays a twist-boat conformation. The 1,4-dihydro­pyridine ring is approximately perpendicular to the benzene ring [dihedral angle = 88.3 (1)°]. The mol­ecular conformation is stabilized by an intra­molecular N—H⋯O hydrogen bond. In the crystal, mol­ecules are linked by N—H⋯O inter­actions into a *C*(8) chain along [100].

## Related literature

For a related structure, see: Tian *et al.* (2009 ▶). For puckering parameters, see: Cremer & Pople 1(975). For background to neonicotinoid insecticides, see: Mori *et al.* (2001 ▶); Kagabu (1997 ▶); Tian *et al.* (2007 ▶); Jeschke & Nauen (2008 ▶); Tomizawa & Casida (2005 ▶). For set-graph notation, see: Bernstein *et al.* (1995 ▶). For puckering parameters, see: Cremer & Pople (1975 ▶);
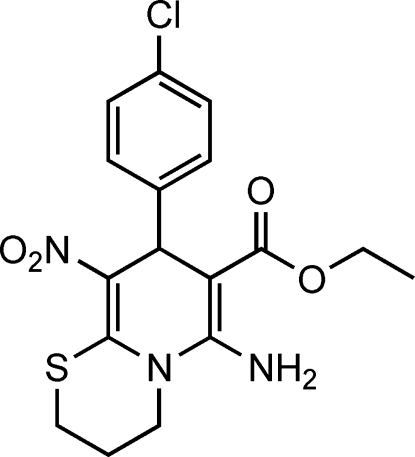


## Experimental

### 

#### Crystal data

C_17_H_18_ClN_3_O_4_S
*M* *_r_* = 395.85
Triclinic, 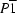

*a* = 8.6376 (8) Å
*b* = 9.9719 (8) Å
*c* = 12.0616 (11) Å
α = 110.970 (8)°
β = 103.252 (8)°
γ = 99.663 (7)°
*V* = 907.88 (16) Å^3^
*Z* = 2
Mo *K*α radiation
μ = 0.35 mm^−1^
*T* = 298 K
0.58 × 0.28 × 0.16 mm

#### Data collection

Bruker APEXII CCD area-detector diffractometer
Absorption correction: multi-scan (*SADABS*; Bruker, 2005 ▶) *T*_min_ = 0.821, *T*_max_ = 0.946
7457 measured reflections
3692 independent reflections
2856 reflections with *I* > 2σ(*I*)
*R*_int_ = 0.021

#### Refinement

*R*[*F* ^2^ > 2σ(*F* ^2^)] = 0.046
*wR*(*F* ^2^) = 0.119
*S* = 1.03
3692 reflections
242 parameters
4 restraints
H-atom parameters constrained
Δρ_max_ = 0.30 e Å^−3^
Δρ_min_ = −0.38 e Å^−3^

### 

Data collection: *APEX2* (Bruker, 2005 ▶); cell refinement: *SAINT* (Bruker, 2005 ▶); data reduction: *SAINT*; program(s) used to solve structure: *SIR97* (Altomare *et al.*, 1999 ▶); program(s) used to refine structure: *SHELXL97* (Sheldrick, 2008 ▶); molecular graphics: *SHELXTL* (Sheldrick, 2008 ▶); software used to prepare material for publication: *WinGX* (Farrugia, 1999 ▶).

## Supplementary Material

Crystal structure: contains datablock(s) I, global. DOI: 10.1107/S1600536811032193/bx2362sup1.cif

Structure factors: contains datablock(s) I. DOI: 10.1107/S1600536811032193/bx2362Isup2.hkl

Supplementary material file. DOI: 10.1107/S1600536811032193/bx2362Isup3.cml

Additional supplementary materials:  crystallographic information; 3D view; checkCIF report

## Figures and Tables

**Table 1:** Hydrogen-bond geometry (Å, °)

*D*—H⋯*A*	*D*—H	H⋯*A*	*D*⋯*A*	*D*—H⋯*A*
N2—H2*B*⋯O1	0.89 (4)	1.95 (5)	2.680 (4)	138 (4)
N2—H2*A*⋯O4^i^	0.87 (4)	2.16 (4)	2.850 (3)	137 (4)

Symmetry code: (i) 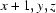.

**Fig. 1.**
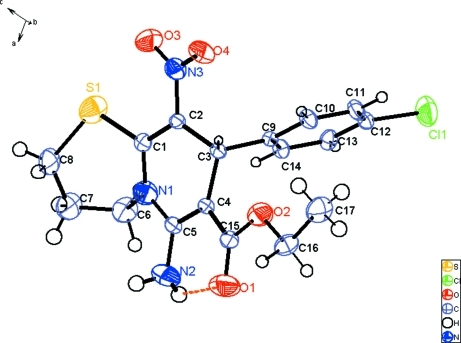
The molecular structure of the title compound with atom numbering scheme. Displacement ellipsoids are drawn at the 30% probability level. The H atoms are shown as spheres of arbitrary size. The hydrogen bond is showing as dashed lines.

## supplementary crystallographic information

### Comment

Neonicotinoids, represented by imidacloprid (IMI) are extensively utilized as systemic insecticides for crop protection against piercing-sucking insect pests, currently accounting for over one-fifth of the world insecticide market (Jeschke *et al.*, 2008). Neonicotinoids act as selective agonists at the insect nicotinic acetylcholine receptor (nAChR), combining excellent insecticidal effectiveness with minimal risk to people and wildlife (Tomizawa *et al.*, 2005). Our interest was introducing sulfur atom into the lead struture and synthesizing a series of new compounds, in which the title compound exhibited moderate insecticidal activities against pea aphids.

Structure of the title compound is shown in Fig. 1 with atom-numbering scheme. the thiazinane ring displays a twist-boat conformation with puckering parameters Q_T_= 0.771 (3)Å, θ= 93.0 (2)°, φ= 156.9 (3)° (Cremer & Pople, 1975). The 1,4-dihydropyridine ring is almost plane conformation and aproximately perpendicular to the phenyl ring ( dihedral angle of 88.3 (1)°). The molecular conformation is stabilized by one intramolecular N—H···O hydrogen bond. The molecular conformation is stabilized by one intramolecular N—H···O hydrogen bond. The molecules are linked by N—H···O interactions, into a chain along [100] with graph-set notation *C*(8) (Bernstein *et al.*,1995), Table 1, Fig. 2.

### Experimental

A solution of Ethyl cyanoacetate (15 mmol) in anhydrous alcohol (15 ml) was added dropwise to a solution of 4-Chlorobenzaldehyde (15 mmol) in anhydrous alcohol (15 ml) at room temperature. After 5 min of stirring at room temperature, piperidine (0.1 mmol) used as catalyst was added dropwise. The resulting mixture was stirred for another 2 h, then (*Z*)-2- (nitromethylene)-1,3-thiazinane (10 mmol) was added to the reaction mixture, refluxed for 15–20 h, and cooled to room temperature. Solid crystal products was filtered, washed with CH_2_Cl_2_, and dried to give desired products. Single crystals suitable for X-ray analysis were obtained by slow evaporation of a solution of dichloromethane and ethyl acetate of the title compound.

### Refinement

All H atoms were placed in their calculated positions and then refined using riding model with C—H = 0.93–0.97 Å, *U*_iso_(H) = 1.2 (1.5 for methyl groups) times *U*_eq_(C).

### Crystal data

**Table:**

C_17_H_18_ClN_3_O_4_S	*Z* = 2
*M**_r_* = 395.85	*F*(000) = 412
Triclinic, *P*1	*D*_x_ = 1.448 Mg m^−^^3^
Hall symbol: -P 1	Mo *K*α radiation, λ = 0.71073 Å
*a* = 8.6376 (8) Å	Cell parameters from 4541 reflections
*b* = 9.9719 (8) Å	θ = 2.4–27.7°
*c* = 12.0616 (11) Å	µ = 0.35 mm^−^^1^
α = 110.970 (8)°	*T* = 298 K
β = 103.252 (8)°	Prism, colourless
γ = 99.663 (7)°	0.58 × 0.28 × 0.16 mm
*V* = 907.88 (16) Å^3^	

### Data collection

**Table:**

Bruker APEXII CCD area-detector diffractometer	3692 independent reflections
Radiation source: fine-focus sealed tube	2856 reflections with *I* > 2σ(*I*)
graphite	*R*_int_ = 0.021
φ and ω scans	θ_max_ = 26.4°, θ_min_ = 3.4°
Absorption correction: multi-scan (*SADABS*; Bruker, 2005)	*h* = −10→10
*T*_min_ = 0.821, *T*_max_ = 0.946	*k* = −12→12
7457 measured reflections	*l* = −15→14

### Refinement

**Table:**

Refinement on *F*^2^	Primary atom site location: structure-invariant direct methods
Least-squares matrix: full	Secondary atom site location: difference Fourier map
*R*[*F*^2^ > 2σ(*F*^2^)] = 0.046	Hydrogen site location: inferred from neighbouring sites
*wR*(*F*^2^) = 0.119	H-atom parameters constrained
*S* = 1.03	*w* = 1/[σ^2^(*F*_o_^2^) + (0.0499*P*)^2^ + 0.3496*P*] where *P* = (*F*_o_^2^ + 2*F*_c_^2^)/3
3692 reflections	(Δ/σ)_max_ < 0.001
242 parameters	Δρ_max_ = 0.30 e Å^−^^3^
4 restraints	Δρ_min_ = −0.38 e Å^−^^3^

### Special details

**Table:**

Geometry. All e.s.d.'s (except the e.s.d. in the dihedral angle between two l.s. planes) are estimated using the full covariance matrix. The cell e.s.d.'s are taken into account individually in the estimation of e.s.d.'s in distances, angles and torsion angles; correlations between e.s.d.'s in cell parameters are only used when they are defined by crystal symmetry. An approximate (isotropic) treatment of cell e.s.d.'s is used for estimating e.s.d.'s involving l.s. planes.
Refinement. Refinement of *F*^2^ against ALL reflections. The weighted *R*-factor *wR* and goodness of fit *S* are based on *F*^2^, conventional *R*-factors *R* are based on *F*, with *F* set to zero for negative *F*^2^. The threshold expression of *F*^2^ > σ(*F*^2^) is used only for calculating *R*-factors(gt) *etc*. and is not relevant to the choice of reflections for refinement. *R*-factors based on *F*^2^ are statistically about twice as large as those based on *F*, and *R*- factors based on ALL data will be even larger.

### Fractional atomic coordinates and isotropic or equivalent isotropic displacement parameters (Å^2^)

**Table:**

	*x*	*y*	*z*	*U*_iso_*/*U*_eq_	
S1	0.55649 (9)	0.69346 (7)	1.02703 (6)	0.0526 (2)	
Cl1	−0.06895 (8)	0.51657 (8)	0.27303 (6)	0.0582 (2)	
O1	0.7918 (2)	1.0629 (2)	0.70241 (19)	0.0661 (6)	
C12	0.0733 (3)	0.6242 (3)	0.4217 (2)	0.0402 (5)	
C13	0.1908 (3)	0.5640 (2)	0.4697 (2)	0.0426 (5)	
H13	0.1918	0.4665	0.4251	0.051*	
C10	0.1846 (3)	0.8525 (3)	0.6042 (2)	0.0415 (5)	
H10	0.1810	0.9488	0.6496	0.050*	
C15	0.6594 (3)	1.0300 (3)	0.7201 (2)	0.0440 (5)	
C14	0.3069 (3)	0.6519 (2)	0.5857 (2)	0.0385 (5)	
H14	0.3874	0.6128	0.6183	0.046*	
C11	0.0684 (3)	0.7665 (3)	0.4875 (2)	0.0478 (6)	
H11	−0.0120	0.8051	0.4543	0.057*	
C16	0.5733 (4)	1.1999 (3)	0.6395 (3)	0.0589 (7)	
H16A	0.6017	1.1547	0.5639	0.071*	
H16B	0.6655	1.2843	0.6994	0.071*	
C6	0.7619 (4)	0.6382 (3)	0.8644 (3)	0.0706 (8)	
H6A	0.8354	0.6341	0.8141	0.085*	
H6B	0.6767	0.5437	0.8247	0.085*	
C5	0.7196 (3)	0.8499 (3)	0.8047 (2)	0.0405 (5)	
O2	0.53736 (19)	1.09179 (17)	0.69114 (15)	0.0449 (4)	
O3	0.2722 (2)	0.75602 (19)	0.98083 (15)	0.0513 (4)	
O4	0.21057 (18)	0.91022 (19)	0.89999 (15)	0.0484 (4)	
C3	0.4386 (2)	0.8934 (2)	0.78073 (18)	0.0317 (4)	
H3	0.4147	0.9901	0.8135	0.038*	
N3	0.3003 (2)	0.8315 (2)	0.92150 (16)	0.0373 (4)	
C9	0.3064 (2)	0.7963 (2)	0.65422 (18)	0.0312 (4)	
C1	0.5529 (3)	0.7644 (2)	0.91386 (19)	0.0347 (5)	
N1	0.6833 (2)	0.7590 (2)	0.86615 (17)	0.0422 (4)	
C4	0.6108 (2)	0.9223 (2)	0.76932 (19)	0.0354 (5)	
C2	0.4350 (2)	0.8266 (2)	0.87397 (18)	0.0311 (4)	
C8	0.7765 (4)	0.7224 (4)	1.0854 (3)	0.0815 (10)	
H8A	0.8287	0.8291	1.1280	0.098*	
H8B	0.7976	0.6811	1.1472	0.098*	
N2	0.8663 (3)	0.8554 (3)	0.7814 (2)	0.0656 (7)	
H2A	0.943 (3)	0.848 (4)	0.8361	0.068*	
H2B	0.895 (4)	0.923 (4)	0.756	0.068*	
C17	0.4207 (4)	1.2497 (4)	0.6110 (3)	0.0777 (9)	
H17A	0.3289	1.1643	0.5560	0.117*	
H17B	0.4372	1.3166	0.5713	0.117*	
H17C	0.3982	1.3001	0.6873	0.117*	
C7	0.8573 (5)	0.6566 (5)	0.9903 (4)	0.0968 (12)	
H7A	0.9679	0.7208	1.0146	0.116*	
H7B	0.8672	0.5600	0.9875	0.116*	

### Atomic displacement parameters (Å^2^)

**Table:**

	*U*^11^	*U*^22^	*U*^33^	*U*^12^	*U*^13^	*U*^23^
S1	0.0649 (4)	0.0527 (4)	0.0599 (4)	0.0256 (3)	0.0266 (3)	0.0369 (3)
Cl1	0.0493 (4)	0.0660 (4)	0.0387 (3)	0.0006 (3)	−0.0040 (3)	0.0164 (3)
O1	0.0412 (10)	0.0883 (14)	0.0853 (14)	0.0094 (9)	0.0283 (10)	0.0529 (12)
C12	0.0315 (11)	0.0478 (13)	0.0337 (11)	−0.0002 (9)	0.0041 (9)	0.0173 (10)
C13	0.0494 (13)	0.0377 (11)	0.0354 (12)	0.0073 (10)	0.0095 (10)	0.0139 (10)
C10	0.0344 (12)	0.0419 (12)	0.0434 (12)	0.0143 (9)	0.0070 (10)	0.0138 (10)
C15	0.0362 (12)	0.0493 (13)	0.0399 (12)	0.0006 (10)	0.0100 (10)	0.0172 (10)
C14	0.0364 (11)	0.0411 (12)	0.0380 (12)	0.0132 (9)	0.0067 (10)	0.0184 (10)
C11	0.0335 (12)	0.0581 (15)	0.0487 (14)	0.0150 (11)	0.0029 (11)	0.0238 (12)
C16	0.0721 (18)	0.0534 (15)	0.0672 (17)	0.0148 (13)	0.0333 (15)	0.0368 (14)
C6	0.087 (2)	0.0748 (19)	0.076 (2)	0.0578 (17)	0.0406 (18)	0.0346 (16)
C5	0.0283 (11)	0.0528 (13)	0.0365 (11)	0.0099 (10)	0.0089 (9)	0.0153 (10)
O2	0.0443 (9)	0.0480 (9)	0.0495 (9)	0.0093 (7)	0.0181 (8)	0.0274 (8)
O3	0.0466 (10)	0.0598 (10)	0.0512 (10)	0.0043 (8)	0.0218 (8)	0.0277 (9)
O4	0.0303 (8)	0.0637 (10)	0.0541 (10)	0.0190 (8)	0.0145 (8)	0.0241 (9)
C3	0.0271 (10)	0.0319 (10)	0.0333 (10)	0.0069 (8)	0.0085 (9)	0.0114 (8)
N3	0.0285 (9)	0.0408 (10)	0.0320 (9)	0.0012 (8)	0.0060 (8)	0.0091 (8)
C9	0.0250 (10)	0.0370 (11)	0.0313 (10)	0.0058 (8)	0.0086 (8)	0.0151 (9)
C1	0.0381 (11)	0.0283 (10)	0.0327 (11)	0.0073 (9)	0.0098 (9)	0.0085 (8)
N1	0.0406 (10)	0.0479 (11)	0.0440 (11)	0.0222 (9)	0.0157 (9)	0.0196 (9)
C4	0.0256 (10)	0.0430 (11)	0.0332 (11)	0.0049 (9)	0.0076 (9)	0.0141 (9)
C2	0.0262 (10)	0.0330 (10)	0.0294 (10)	0.0050 (8)	0.0082 (8)	0.0096 (8)
C8	0.079 (2)	0.125 (3)	0.073 (2)	0.060 (2)	0.0246 (18)	0.062 (2)
N2	0.0324 (11)	0.106 (2)	0.0749 (17)	0.0273 (12)	0.0228 (11)	0.0482 (15)
C17	0.092 (2)	0.082 (2)	0.093 (2)	0.0385 (19)	0.039 (2)	0.059 (2)
C7	0.095 (3)	0.154 (4)	0.104 (3)	0.088 (3)	0.049 (2)	0.088 (3)

### Geometric parameters (Å, °)

**Table:**

S1—C1	1.744 (2)	C6—H6B	0.9700
S1—C8	1.802 (3)	C5—N2	1.356 (3)
Cl1—C12	1.745 (2)	C5—C4	1.361 (3)
O1—C15	1.224 (3)	C5—N1	1.400 (3)
C12—C11	1.369 (3)	O3—N3	1.242 (2)
C12—C13	1.382 (3)	O4—N3	1.242 (2)
C13—C14	1.384 (3)	C3—C2	1.502 (3)
C13—H13	0.9300	C3—C4	1.514 (3)
C10—C11	1.387 (3)	C3—C9	1.531 (3)
C10—C9	1.390 (3)	C3—H3	0.9800
C10—H10	0.9300	N3—C2	1.410 (3)
C15—O2	1.347 (3)	C1—C2	1.362 (3)
C15—C4	1.449 (3)	C1—N1	1.378 (3)
C14—C9	1.382 (3)	C8—C7	1.488 (5)
C14—H14	0.9300	C8—H8A	0.9700
C11—H11	0.9300	C8—H8B	0.9700
C16—O2	1.450 (3)	N2—H2A	0.855 (17)
C16—C17	1.495 (4)	N2—H2B	0.859 (17)
C16—H16A	0.9700	C17—H17A	0.9600
C16—H16B	0.9700	C17—H17B	0.9600
C6—N1	1.475 (3)	C17—H17C	0.9600
C6—C7	1.482 (4)	C7—H7A	0.9700
C6—H6A	0.9700	C7—H7B	0.9700
			
C1—S1—C8	98.75 (13)	C9—C3—H3	107.7
C11—C12—C13	121.3 (2)	O4—N3—O3	121.78 (18)
C11—C12—Cl1	119.83 (18)	O4—N3—C2	118.18 (17)
C13—C12—Cl1	118.85 (18)	O3—N3—C2	120.02 (18)
C12—C13—C14	118.5 (2)	C14—C9—C10	118.33 (19)
C12—C13—H13	120.7	C14—C9—C3	120.93 (18)
C14—C13—H13	120.7	C10—C9—C3	120.73 (19)
C11—C10—C9	120.8 (2)	C2—C1—N1	119.40 (19)
C11—C10—H10	119.6	C2—C1—S1	124.77 (16)
C9—C10—H10	119.6	N1—C1—S1	115.80 (16)
O1—C15—O2	121.5 (2)	C1—N1—C5	120.12 (18)
O1—C15—C4	126.6 (2)	C1—N1—C6	116.9 (2)
O2—C15—C4	111.94 (18)	C5—N1—C6	122.2 (2)
C9—C14—C13	121.7 (2)	C5—C4—C15	119.96 (19)
C9—C14—H14	119.2	C5—C4—C3	121.27 (19)
C13—C14—H14	119.2	C15—C4—C3	118.76 (19)
C12—C11—C10	119.4 (2)	C1—C2—N3	119.81 (18)
C12—C11—H11	120.3	C1—C2—C3	123.84 (18)
C10—C11—H11	120.3	N3—C2—C3	116.35 (18)
O2—C16—C17	107.0 (2)	C7—C8—S1	116.0 (3)
O2—C16—H16A	110.3	C7—C8—H8A	108.3
C17—C16—H16A	110.3	S1—C8—H8A	108.3
O2—C16—H16B	110.3	C7—C8—H8B	108.3
C17—C16—H16B	110.3	S1—C8—H8B	108.3
H16A—C16—H16B	108.6	H8A—C8—H8B	107.4
N1—C6—C7	113.6 (3)	C5—N2—H2A	115 (2)
N1—C6—H6A	108.8	C5—N2—H2B	114 (2)
C7—C6—H6A	108.8	H2A—N2—H2B	116 (3)
N1—C6—H6B	108.8	C16—C17—H17A	109.5
C7—C6—H6B	108.8	C16—C17—H17B	109.5
H6A—C6—H6B	107.7	H17A—C17—H17B	109.5
N2—C5—C4	124.0 (2)	C16—C17—H17C	109.5
N2—C5—N1	115.0 (2)	H17A—C17—H17C	109.5
C4—C5—N1	121.05 (19)	H17B—C17—H17C	109.5
C15—O2—C16	116.12 (18)	C6—C7—C8	111.6 (3)
C2—C3—C4	109.50 (16)	C6—C7—H7A	109.3
C2—C3—C9	111.93 (15)	C8—C7—H7A	109.3
C4—C3—C9	112.25 (16)	C6—C7—H7B	109.3
C2—C3—H3	107.7	C8—C7—H7B	109.3
C4—C3—H3	107.7	H7A—C7—H7B	108.0
			
C11—C12—C13—C14	−1.3 (3)	C7—C6—N1—C5	120.1 (3)
Cl1—C12—C13—C14	177.68 (16)	N2—C5—C4—C15	−9.6 (4)
C12—C13—C14—C9	0.8 (3)	N1—C5—C4—C15	172.29 (19)
C13—C12—C11—C10	0.6 (3)	N2—C5—C4—C3	170.7 (2)
Cl1—C12—C11—C10	−178.39 (17)	N1—C5—C4—C3	−7.4 (3)
C9—C10—C11—C12	0.6 (3)	O1—C15—C4—C5	2.2 (4)
O1—C15—O2—C16	−0.7 (3)	O2—C15—C4—C5	−179.4 (2)
C4—C15—O2—C16	−179.22 (19)	O1—C15—C4—C3	−178.1 (2)
C17—C16—O2—C15	178.3 (2)	O2—C15—C4—C3	0.3 (3)
C13—C14—C9—C10	0.4 (3)	C2—C3—C4—C5	20.6 (3)
C13—C14—C9—C3	−178.31 (18)	C9—C3—C4—C5	−104.3 (2)
C11—C10—C9—C14	−1.1 (3)	C2—C3—C4—C15	−159.09 (18)
C11—C10—C9—C3	177.56 (19)	C9—C3—C4—C15	76.0 (2)
C2—C3—C9—C14	−61.2 (2)	N1—C1—C2—N3	179.49 (17)
C4—C3—C9—C14	62.4 (2)	S1—C1—C2—N3	−2.5 (3)
C2—C3—C9—C10	120.2 (2)	N1—C1—C2—C3	−0.9 (3)
C4—C3—C9—C10	−116.2 (2)	S1—C1—C2—C3	177.04 (15)
C8—S1—C1—C2	−152.2 (2)	O4—N3—C2—C1	168.14 (18)
C8—S1—C1—N1	25.8 (2)	O3—N3—C2—C1	−13.3 (3)
C2—C1—N1—C5	17.0 (3)	O4—N3—C2—C3	−11.5 (3)
S1—C1—N1—C5	−161.12 (16)	O3—N3—C2—C3	167.08 (17)
C2—C1—N1—C6	−153.1 (2)	C4—C3—C2—C1	−16.8 (3)
S1—C1—N1—C6	28.7 (3)	C9—C3—C2—C1	108.4 (2)
N2—C5—N1—C1	168.8 (2)	C4—C3—C2—N3	162.84 (16)
C4—C5—N1—C1	−12.9 (3)	C9—C3—C2—N3	−72.0 (2)
N2—C5—N1—C6	−21.6 (3)	C1—S1—C8—C7	−54.3 (3)
C4—C5—N1—C6	156.7 (2)	N1—C6—C7—C8	36.1 (5)
C7—C6—N1—C1	−70.0 (3)	S1—C8—C7—C6	25.0 (5)

### Hydrogen-bond geometry (Å, °)

**Table:**

*D*—H···*A*	*D*—H	H···*A*	*D*···*A*	*D*—H···*A*
N2—H2B···O1	0.89 (4)	1.95 (5)	2.680 (4)	138 (4)
N2—H2A···O4^i^	0.87 (4)	2.16 (4)	2.850 (3)	137 (4)

Symmetry codes: (i) *x*+1, *y*, *z*.
